# Anatomically accurate model of EMG during index finger flexion and abduction derived from diffusion tensor imaging

**DOI:** 10.1371/journal.pcbi.1007267

**Published:** 2019-08-29

**Authors:** Diego Pereira Botelho, Kathleen Curran, Madeleine M. Lowery

**Affiliations:** 1 School of Electrical and Electronic Engineering, University College Dublin, Belfield, Dublin, Ireland; 2 School of Medicine, University College Dublin, Belfield, Dublin, Ireland; University of Virginia, UNITED STATES

## Abstract

This study presents a modelling framework in which information on muscle fiber direction and orientation during contraction is derived from diffusion tensor imaging (DTI) and incorporated in a computational model of the surface electromyographic (EMG) signal. The proposed model makes use of the principle of reciprocity to simultaneously calculate the electric potentials produced at the recording electrode by charges distributed along an arbitrary number of muscle fibers within the muscle, allowing for a computationally efficient evaluation of extracellular motor unit action potentials. The approach is applied to the complex architecture of the first dorsal interosseous (FDI) muscle of the hand to simulate EMG during index finger flexion and abduction. Using diffusion tensor imaging methods, the results show how muscle fiber orientation and curvature in this intrinsic hand muscle change during flexion and abduction. Incorporation of anatomically accurate muscle architecture and other hand tissue morphologies enables the model to capture variations in extracellular action potential waveform shape across the motor unit population and to predict experimentally observed differences in EMG signal features when switching from index finger abduction to flexion. The simulation results illustrate how structural and electrical properties of the tissues comprising the volume conductor, in combination with fiber direction and curvature, shape the detected action potentials. Using the model, the relative contribution of motor units of different sizes located throughout the muscle under both conditions is examined, yielding a prediction of the detection profile of the surface EMG electrode array over the muscle cross-section.

## Author summary

Advances in diffusion tensor imaging are providing new information on muscle architecture and the orientation of muscle fibers *in vivo*. The arrangement of muscle fibers, in combination with geometrical and electrical properties of the surrounding biological tissues, shapes the electrical signal recorded at the skin surface during muscle contraction. As new recording and analysis methods enable muscle and motor unit activity to be examined during complex dynamic contractions, changes in muscle fiber orientation and surrounding tissue properties pose challenges for the interpretation of these data. Here we incorporate details of tissue geometry and muscle fiber architecture obtained using anatomical and diffusion MRI into an anatomically accurate model of electromyography (EMG) signal generation in the first dorsal interosseous muscle of the hand. The new modeling approach presented integrates interdependent electrical and geometrical properties in an anatomically accurate manner, leading to a realistic EMG model where tissue electrical properties are inherently related to bioelectric aspects of muscle activation. The results show how muscle fiber orientation and curvature change according to the direction of force generation, influencing the EMG signal, and provide new insights on how constitutive, anatomical and physiological properties contribute to shape motor unit action potentials detected at the skin surface.

## Introduction

Investigation of the mechanisms underlying muscle activation and their changes under different conditions is heavily reliant on data derived from electromyography (EMG). Electromyographic signals recorded at electrodes on the skin surface—or within the muscle—are composed of temporally-distributed extracellular action potentials produced by the activation of motor units lying within the electrode detection volume. These signals contain a rich array of information on both the motoneuron firing patterns controlling the muscle activation and the properties of the motor units. The timing of individual motor unit action potentials is determined by the discharge times of the corresponding motoneurons, while the shape of the extracellular motor unit action potential is governed by the size and location of the motor unit, the characteristics and arrangement of its fibers, the electrical and geometrical properties of the surrounding tissues and the electrode configuration and position. As the influence of each of these factors can be difficult to discern through experimentation alone, mathematical modelling has become an established tool to assist in the interpretation of EMG.

Volume conductor models describe the geometrical and electrical properties of the tissues in which the electrical sources and electrodes are located and the resulting distribution of electric fields and current within these tissues. Early volume conductor models were based upon analytical solutions for muscle fibers within infinite or idealized domains based on regular geometries representing the surrounding tissue [1–6]. These models provided insight into the composition of EMG signals, including the effects of fiber location, tissue conductivity and electrode configuration [4,7–9]. However, more realistic scenarios considering the anatomical complexity of different biological tissues, such as muscle, bone, fat, blood vessels and skin, could only be introduced with the implementation of numerical models based on techniques such as the finite element method (FEM) [10–16]. Extending this approach, anatomically accurate numerical models can be derived by incorporating imaging data to create 3D representations of the biological tissues in the region of interest [17,18].

Although numerical models allow for an improved understanding of EMG signal generation and volume conduction, one key element of the problem has yet to be addressed: the realistic estimation of muscle fiber architecture and its changes during activation. Consequently, in most models developed, simulated action potentials propagate along virtual straight muscle fibers [8,19]. The muscle electrical anisotropy, in turn, is substantially simplified, often consisting of electromagnetic properties defined using cylindrical coordinate systems [14,19,20]. Approaches for incorporating curvilinear fibers and electrical anisotropy have been proposed, but they are restricted to resting states [18] or idealized volume conductors [21,22].

In the last decade, diffusion-weighted MRI (DWI) has begun to be applied to study muscle tissue [23–26]. More specifically, diffusion tensor imaging (DTI) has proven to be a powerful technique for quantifying muscle anisotropy, enabling fiber orientation and curvature to be estimated and providing an alternative to cadaveric studies. Indeed, the employment of imaging techniques based on MRI, such as DTI, has provided a means to visualize the architecture of complex and deep muscle structures which cannot otherwise be studied *in vivo*. Furthermore, it provides an opportunity to study changes in architecture related to joint position and muscle activation [27,28].

In this study, we develop an anatomically accurate, subject-specific surface EMG model of the first dorsal interosseous (FDI) muscle of the hand in which muscle fiber arrangement and electrical anisotropy are estimated using DTI fiber tracking. The FDI is a multifunctional muscle solely responsible for index finger abduction and contributing also to flexion, thus playing an important role in precise movements of the hand [29]. Cortico-motoneurons are known to project directly to the motoneuron pool of the FDI [30], making it a strategic target for investigation of corticospinal activity or fine motor control under normal and pathological conditions. Though previous studies have utilized DTI to investigate the properties of upper and lower limb muscles [23,31], this is the first time the technique has been used to examine intrinsic muscles of the hand. The model is used to understand how volume conductor structural and electrical properties, in combination with fiber orientation and curvature, determine the EMG signal features, and to predict the contribution to the surface-detected signal from motor units of different sizes located throughout the muscle cross-section. Understanding the complex interaction and influence of these factors is critical in enabling accurate information to be reliably extracted from the surface EMG signal.

## Methods

### Ethics statement

This study was approved by the Human Sciences Research Ethics Committee at University College Dublin. Written informed consent was obtained from the participant.

### Anatomical EMG model

**Fig 1:**
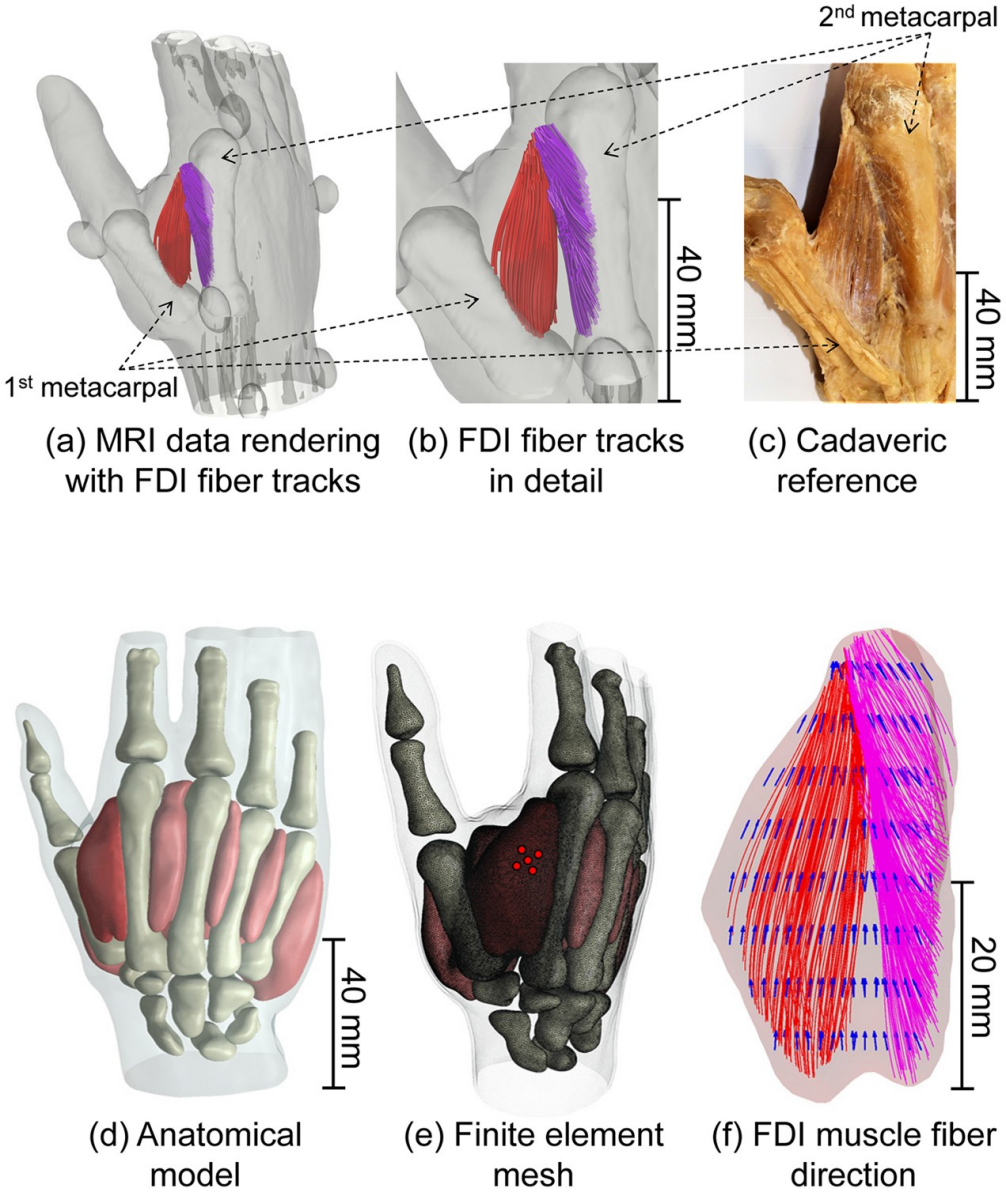
3D models derived from MRI/DWI data with reference cadaveric specimen. (a) Isosurface rendering of the MRI data with fiber tracks of the superficial (red) and deep (pink) heads of the FDI muscle at rest (the positions of the first and second metacarpals are highlighted). Elliptical objects around the subject’s hand are oil capsules utilized as geometrical reference. (b) Closer view of muscle fiber tracks of both heads of the FDI. (c) Superficial and deep heads of the FDI muscle in a cadaveric specimen. Note consistency in fiber direction, origin, and insertion in both superficial and deep heads of the muscle in the DTI derived image and the cadaveric specimen. (d) Segmented hand model derived from MRI data. (e) Finite element discretization of the hand model (tetrahedral mesh). EMG electrodes used in simulations and experimental recordings are indicated by the red discs. (f) The FDI muscle (beige) is presented with the muscle fiber tracks derived from the DTI analysis (red and pink) and the vector field (blue) generated from the interpolation/extrapolation of these tracks directions over the entire FDI volume. This vector field determines the direction of highest electrical conductivity and the trajectories of virtual muscle fibers within the FDI.

A detailed 3D model of the hand at rest was developed based on anatomical MRI data (Fig 1d). Muscle fiber direction and curvature within the FDI muscle at rest, and during index finger abduction and flexion were estimated from the corresponding diffusion MRI data using deterministic fiber tracking (Fig 1a and 1b). Extracellular potentials generated at the electrode during motor unit activation, known as motor unit action potentials (MUAPs), and surface EMG signals were simulated for a population of motor units within the muscle and compared with experimental data recorded from the same subject.

### Motor unit action potentials

The propagation of action potentials along the muscle fibers comprising each motor unit yields a time-dependent extracellular potential distribution throughout the biological tissues surrounding the fiber. From an electrical perspective, this volume conductor can be considered linear, as the extracellular potential produced by the motor unit activation can be described as the superposition of the time-dependent extracellular potentials, *φ*_*ec*_, produced by the propagation of individual action potentials along single muscle fibers which can be estimated as [3]:

$$\varphi_{ec}(x,t)=i_{0}(t)*h(x,t),$$ (1)

where **x** is the spatial coordinate, *t* is time, *i*_0_(*t*) represents the intracellular transmembrane current and *h*(**x**,*t*) is the extracellular potential at **x** produced by a unitary point current source propagating along the muscle fiber at conduction velocity *u*. *h*(**x**,*t*) is thus a weighting function representing the impulse response of such system at a given observation point **x**. In the EMG model presented, these weighting functions are computed numerically by means of a finite element resolution of the electric potential distribution within the subject’s hand. Taking advantage of the linear nature of the problem and the reciprocity principle, we evaluate *h*(**x**,*t*) as the potential distribution along the muscle fibers produced by a unit source at the electrode. This allows us to solve the electromagnetic problem simultaneously for an arbitrary number of muscle fibers just once per electrode. The weighting functions are then evaluated in a post-processing step by computing the potentials along the individual fibers. This approach results in a novel and computationally efficient way of simulating EMG signals generated by a very large number of muscle fibers.

The transmembrane current *i*_0_(*t*), was derived from the analytical description of the transmembrane potential (mV) formulated by Rosenfalck [32]:

$$v_{m}(l)=96{\left(\alpha .l\right)}^{3}e^{-\alpha .l}-90,$$ (2)

where *l* is distance along the fiber in mm and *α* is a scaling factor allowing adjustment of the action potential length. An intermediate variable, the transmembrane current per unit length, *i*_*m*_(*l*), in A/mm^2^, is related to the second spatial derivative of the transmembrane potential, *v*_*m*_(*l*), as follows [33]:

$$i_{m}(l)=\sigma_{ic}\pi r^{2}\frac{d^{2}v_{m}(l)}{{dl}^{2}},$$ (3)

where *r* is the muscle fiber radius and *σ*_*ic*_ the intracellular conductivity. Finally, assuming that the conduction velocity remains constant along the muscle fiber, the time-dependent transmembrane current *i*_0_(*t*) can be expressed as:

$$i_{0}(t)=i_{m}(ut)\frac{dl}{dt}=i_{m}(ut).u.$$ (4)

To ensure charge balance during generation and extinction of action potentials at the neuromuscular and musculotendon junctions, an approach based on that proposed by Dimitrova [34,35] and Plonsey [33] was implemented. This approach consists in the imposition of stationary compensatory current sources at the fiber ends and innervation point to satisfy the condition that at each point in time the sum of currents along the fiber remains equal to zero.

### Finite element model

The weighting functions *h*(**x**,*t*) were reciprocally evaluated using a finite element model of the subject’s hand. MRI data for the hand at rest were first segmented into distinct tissues: skin, fat, cancellous bone, cortical bone, and muscle (Fig 1d). After segmentation, the hand model was discretized into a finite element tetrahedral mesh with a fine mesh resolution imposed on areas surrounding the FDI, and a coarser mesh used elsewhere (Fig 1e). The segmentation and meshing procedures were performed using scanIP (Synopsys, Inc.). The meshed hand geometry was then exported to COMSOL (COMSOL, Inc.), where the subsequent steps required for the resolution of the electromagnetic problem were carried out.

Assuming that wave propagation, as well as inductive and capacitive effects may be neglected [36], the mathematical formulation of the problem can be derived from the basic principle of electric current conservation (electrokinetics [37]). Thus, the static electric potential distribution, *φ*(**x**), produced within the domain by an electric current source applied to a given electrode can be modeled as:

∇⋅σ(x)∇φ(x)=0, (5)

where *σ*(**x**) is the electric conductivity. Eq (5) was implemented in COMSOL in its weak form [37].

Making use of the principle of reciprocity, the current sources in the model were applied at the electrode surfaces, rather than the muscle fibers. On these surfaces, the following relation holds:

$$\sigma (x)\nabla \varphi (x)\cdot n(x)=J_{s},$$ (6)

where **n**(**x**) is the unitary normal vector directed outwards on the domain boundary and *J*_*s*_ is the superficial density of electric current imposed at the electrode contact. On the boundaries where the geometric model was truncated, *e*.*g*. the wrist, electric current is allowed to escape the domain, representing currents that would be conducted away from the hand into the body. On these interfaces, a ground condition was imposed, *i*.*e*. *φ(****x****)* = 0. All the remaining external boundaries of the domain correspond to the interface between skin and air where it is assumed that no electric current flow is allowed, hence

σ(x)∇φ(x)⋅n(x)=0. (7)

The material properties defined for each tissue were the estimated electric conductivities at 150 Hz, representative of the median frequency of typical surface EMG signals, detailed in Table 1 [38,39].

**Table 1:** Details of finite element model.

**Discretization parameters**	
Number of degrees of freedom (dof)	9,337,906
Number of elements in the FDI	1,945,197
Electric potential approximation order	Quadratic
**Electrical conductivities at 150 Hz** [38,39]	
Skin	4.88x10^-4^ S/m
Fat	4.07x10^-2^ S/m
Cancellous bone	7.56x10^-2^ S/m
Cortical bone	2.00x10^-2^ S/m
Muscle isotropic	0.28 S/m
Muscle longitudinal	0.40 S/m
Muscle transversal	0.09 S/m

Tissue anisotropy within the FDI volume was taken into account through the implementation of a local coordinate system one of whose axes was defined to align with the muscle fiber direction. The estimated muscle fiber direction for any given point in the FDI was obtained from a natural neighbor interpolation [40] of the directions of the muscle tracks obtained from the DTI fiber tracking. In this way, for each point within the FDI, an electrical conductivity tensor was first created in terms of the local coordinate system and then mapped to the global system through a simple coordinate transformation.

The finite element model yielded a linear system with approximately 9 million degrees of freedom that was required to be solved five times for a full simulation, once for each electrode of the EMG array utilized. The resolution of the linear system was carried out through an iterative conjugate gradients solver using a computer equipped with a processor Intel Core i7-6700k and 32 GB of RAM. In average, the solution time for a full simulation was approximately 1 hour. Details of the finite element model are provided in Table 1.

### First dorsal interosseous model

**Fig 2:**
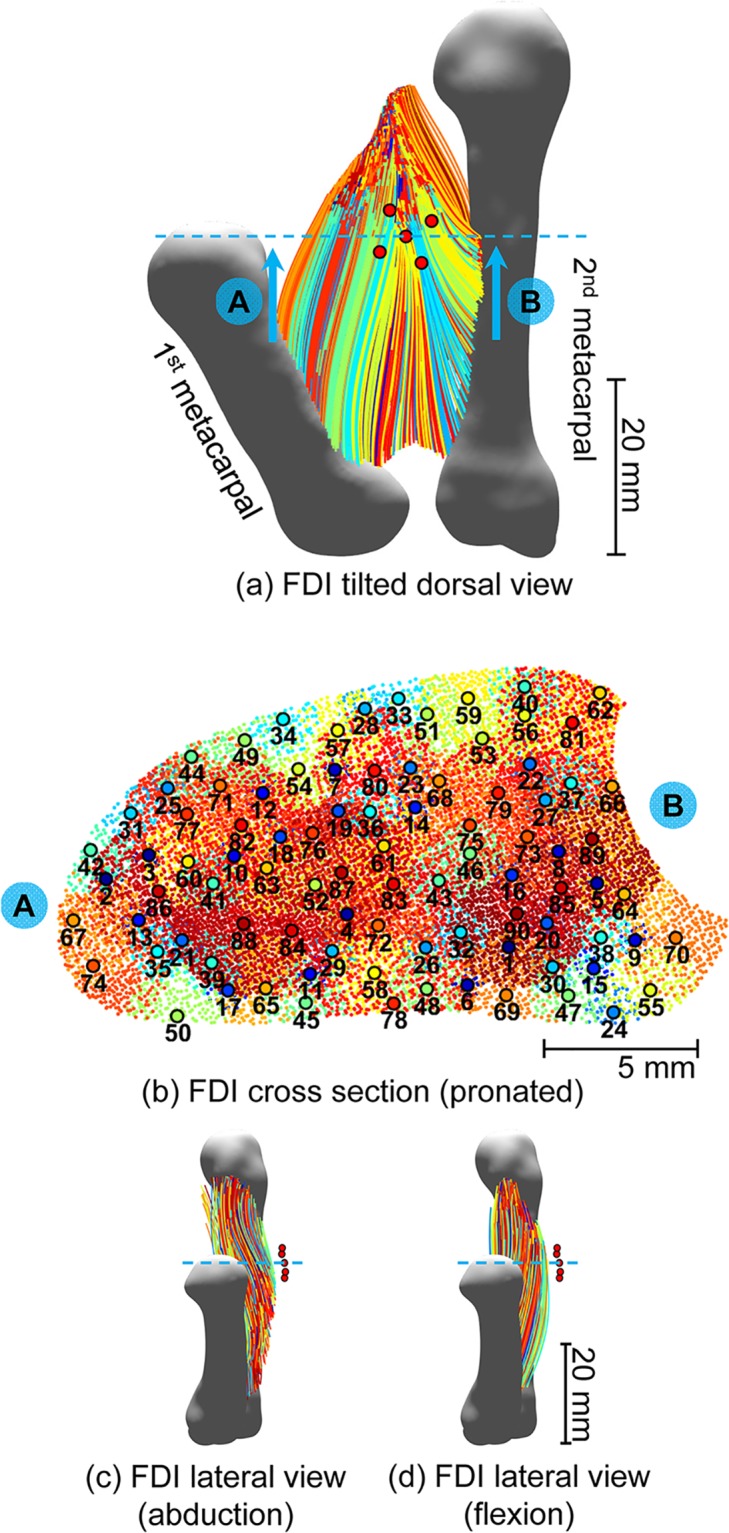
Model of the FDI muscle fibers. (a) Dorsal view of the simulated fibers (3D model tilted 35 degrees right). Blue dashed line indicates the position of the cross-section shown in (b) and the red discs illustrate position of the EMG electrodes. (b) FDI cross-section directly under the EMG array: individual fibers crossing the transverse plane are indicated by the colored dots. The centers of motor unit territories (colored discs with black edges) are numbered in order of motor unit recruitment. (c) Lateral view of the FDI muscle fibers model during abduction (radial side). (d) Lateral view of the FDI muscle fibers model during flexion (radial side). FDI muscle fibers shown in (c) and (d) were plotted with a down-sampling rate of 50 for clarity. Fibers belonging to the same motor unit are shown in the same color.

Three detailed models of the FDI muscle were implemented, one for each of the conditions analyzed: rest, index finger abduction and index finger flexion. A cross-section of the FDI on the axial plane was defined to serve as a geometrical support for the generation of virtual muscle fibers whose trajectories were derived from the results of the DTI fiber tracking. In the model presented, this reference plane was chosen to match the position of the center electrode used in the recording of the subject’s electromyogram, transecting the FDI volume just distal to its midline (Fig 2a). Points representing the positions where the virtual muscle fibers cross the reference plane were randomly located throughout the defined FDI cross-section according to a Sobol distribution assuming a uniform muscle fiber density of 350 fibers/mm^2^ (Fig 2b).

The properties of each motor unit, *i*.*e*., fiber diameter, muscle fiber conduction velocity, number of fibers per motor unit and motor unit territory, were determined based on the parameters presented in Table 2 [41–46].

**Table 2:** First dorsal interosseous model parameters.

First Dorsal Interosseous Model Parameters		
Parameter	Value	Reference
Intracellular conductivity (*σ*_*ic*_)	1 S/m	[41]
Conduction velocity range	3.2–5 m/s	[42]
Fiber diameter range	24.8–40.6 μm	[43]
Mean muscle density	350 fb/mm^2^	[43]
Motor unit density range	25–45 fb/mm^2^	[43,44]
Total number of motor units	120	[45]
Innervation number range	21–1,764 fb/MU	[45]
Fibers distribution across motor units^a^	*y*_*i*_ = *y*_1_*e*^[(ln *R*)/*n*] *i*^	[46]
Total number of fibers	~ 40 k	[46]

^a^ in the formula, *n* is the number of motor units, *y*_*i*_ indicates the innervation number of the motor unit *i*, and *R* is the ratio of innervation numbers between the largest and smallest motor units.

The location of the center of the territory of each motor unit was chosen randomly from the set of fiber crossing points generated. Motor unit territories were then defined as circular regions around the center, with cross-sectional areas determined from the fiber density and the number of fibers in the motor unit. For territories truncated on the muscle border, the radius of the circular area within the muscle was increased to compensate the truncated portions. Fiber crossing points created previously were then attributed to the motor units, according to their territorial coverages, yielding a uniform distribution of muscle fibers throughout the muscle cross-section when all FDI motor units are considered.

At each muscle fiber crossing point, a muscle fiber trajectory was constructed extending away from the cross-sectional plane by 0.1 mm spatial increments according to the directions defined by vector field derived from the DTI fiber tracking (Fig 1f). This vector field determines the direction of highest electrical conductivity and the trajectories of virtual muscle fibers within the FDI. Fibers were terminated at the boundary of the muscle volume or whenever the change in direction from one step to another exceeded 15 degrees. Motoneuron innervation points were placed randomly about the center of the fibers, according to a truncated normal distribution with standard deviation of 10% of the fiber length. Muscle fibers in the model at rest and during abduction and flexion are shown in Fig 2a, 2c and 2d.

### EMG simulation

Surface EMG signals were simulated using a set of 90 MUs, which corresponds to the number of motor units recruited to generate a contraction of approximately 25% of MVC, according to a previously developed FDI motor neuron pool model [47]. The innervation numbers for the simulated set ranged from approximately 20 to 600 fibers per motor unit—nearly 15,500 muscle fibers in total. A scaling factor *α* = 0.5 was used in Eq (2) to match the frequency content of experimentally recorded electromyograms. All remaining muscle parameters were defined as summarized in Table 2.

Motor units firing times were simulated using the FDI motor neuron pool model [47] for 25 s long isometric contractions with force increasing/decreasing at a rate of 5% MVC/second at the start and end of the contraction, in accordance with the experimental conditions. Four synthetic double differential channels with the center electrode as reference were created by convolving the motor units firing times with their respective MUAPs. A white Gaussian noise of standard deviation 107.5 dBW (1 Ω reference impedance) was added to the channels to reproduce the baseline noise observed in the experimental recordings.

### Experimental methods

A single healthy female (42 yrs, height 1.70 m, weight 60 kg) with no history of neurological, neuromuscular, or musculoskeletal disorders participated in the experiments. Ethical approval was obtained from the University College Dublin Human Research Ethics Committee. Magnetic resonance and diffusion weighted images of subject’s right (dominant) hand were acquired with the hand at rest and during index finger abduction and flexion. Surface EMG signals from the subject’s FDI muscle were subsequently recorded.

### Magnetic resonance data acquisition

The subject was positioned in a Philips 3T Achieva MRI scanner lying prone with the right arm above the head and the right hand prone and supported on a purpose-built rig within a 16-element transmit/receive radiofrequency knee coil. The MRI compatible rig was designed to provide a comfortable support for the hand, minimize involuntary hand motion during image acquisition and to allow index finger flexion and abduction with a predefined load and displacement (S1 Fig).

The anatomical characteristics of the hand were assessed through a T1 weighted scan of the hand at rest with 0.8 mm isotropic voxel size and 4 signal averages (NSA). Immediately after the structural T1 weighted scan, diffusion data were acquired using an Echo Planar Imaging (EPI) sequence with one b_0_ volume, 16 gradient orientations, b-value = 500 s/mm^2^, isotropic voxel size = 2 mm and NSA = 7. The diffusion images were recorded under three conditions: (1) hand at rest, (2) isometric index finger abduction and (3) isometric index finger flexion. The force applied by the subject was 6.5 N (measurement error: ± 0.1 N) for both abduction and flexion, which represents approximately 25% (± 1.0%) of the subject maximum voluntary contraction (MVC) force for each movement. The signal to noise ratio (SNR) of non-weighted images was estimated as the ratio of the mean signal intensity and its standard deviation over all the acquired b0 volumes [48], resulting in an SNR of approximately 17 dB.

Acquisition time for each image was approximately 5 minutes. A 10 minutes rest period was allowed between scans to reduce the contribution of muscle fatigue.

### Diffusion data processing and fiber tracking

Acquired diffusion data were corrected for Gibbs ringing artifacts and registered (with resampling) to the T1 anatomical reference using an affine registration, correcting for subject motion, eddy currents distortions and EPI deformations [49].

The estimation of the FDI muscle fiber direction and curvature was then performed through deterministic fiber tracking [50], using the fiber tracking parameters given in Table 3. Obtained muscle fiber tracks were fitted to third order polynomial functions [51] and similar tracks were discarded based on a distance threshold of one voxel [52].

**Table 3:** DTI fiber tracking parameters.

DTI fiber tracking parameters	
Fractional anisotropy threshold	0.15
Angular threshold	15°
Step size	0.4 mm
Region of Interest	FDI volume*
Tracks end regions	1^st^ and 2^nd^ metacarpals*

* the muscle and bones volumes were obtained from the segmentation of the MRI data (metacarpals were dilated by 3 pixels).

### Experimental EMG and force recordings

MVC force for the same subject during isometric index finger flexion (27 N) and abduction (25 N) were recorded using a load cell and a customized rig. The subject then performed 3 isometric muscle contractions in both flexion and abduction at 25% MVC for 25 seconds each in the same MRI-compatible rig used during the scans, ramping to the target force at a rate of 5% MVC per second at the start and end of the contraction. The estimated angular displacement of the index finger around the MP joint was 10 degrees for both abduction and flexion. A 5-minute interval between recordings was implemented to avoid muscle fatigue. Surface EMG was recorded from the FDI muscle using the Delsys Precision Decomposition system (Delsys, Inc.). The EMG sensor comprised an array of five cylindrical electrodes (0.5-mm diameter) located at the corners and at the center of a 5 × 5 mm square. Pairwise differentiation of the five electrodes yielded four channels of sEMG signals. The signals were amplified and filtered between 20 Hz and 2 kHz, sampled at 20 kHz and stored for further processing. Single MUAPs were extracted from the surface EMG signals during the post-processing phase using the decomposition algorithms described in [53] (Delsys, version 4.1.1.0). Decomposition of the surface EMG signal yielded the MUAP firing times and corresponding spike trains. MUAPs were then obtained by spike-triggered averaging the experimentally recorded surface EMG signals using the firing times of each motor unit [54].

### Validation with cadaveric specimen

To validate the results of the DTI fiber tracking, the estimated FDI muscle fiber tracks were compared with the fiber architecture in the FDI of a cadaver (age at death: 91 years; height: 183 cm). For this purpose, the right hand was dissected to expose the superficial and deep heads of the FDI muscle, the first and second metacarpals, and the internal and external tendons. This cadaveric study followed the protocol for hand dissection from Grant's Dissector [55]. The fiber arrangements of the superficial and deep heads of the FDI in the cadaveric specimen and the estimated FDI muscle fiber tracks for the subject participating in the MRI and EMG experiments are presented in Fig 1a, 1b and 1c.

## Results

### Bipennate structure of the FDI revealed through DTI fiber tracking

The muscle fiber tracks estimated for the FDI at rest are presented in Fig 1a and 1b. The bipennate structure of the FDI is captured in the DTI fiber tracking, which reveals the two distinct muscle heads, one originating from the first metacarpal, usually termed the superficial head, and the other originating from the second metacarpal, known as deep head. These two heads of the muscle converge to a tendinous sheet that emerges as the external tendon at the FDI distal extremity and inserts on the base of the index finger proximal phalanx [29]. A close agreement in terms of muscle fiber architecture was observed between the estimated fiber tracks and the cadaveric reference in both superficial and deep heads of the muscle, Fig 1c. Tissue diffusivity measurements also agreed well with values verified previously for other muscles [23,24,28], Table 4.

**Table 4:** DTI analysis summary.

FDI diffusivity measurements (resting state)	
Fractional anisotropy (FA)	0.33 (± 0.07)
Axial diffusivity (AD)	1.85 (± 0.29)
Radial diffusivity (RD)	1.09 (± 0.20)

### Geometrical and electrical properties of the surrounding tissues influence MUAP and EMG signal properties

**Fig 3:**
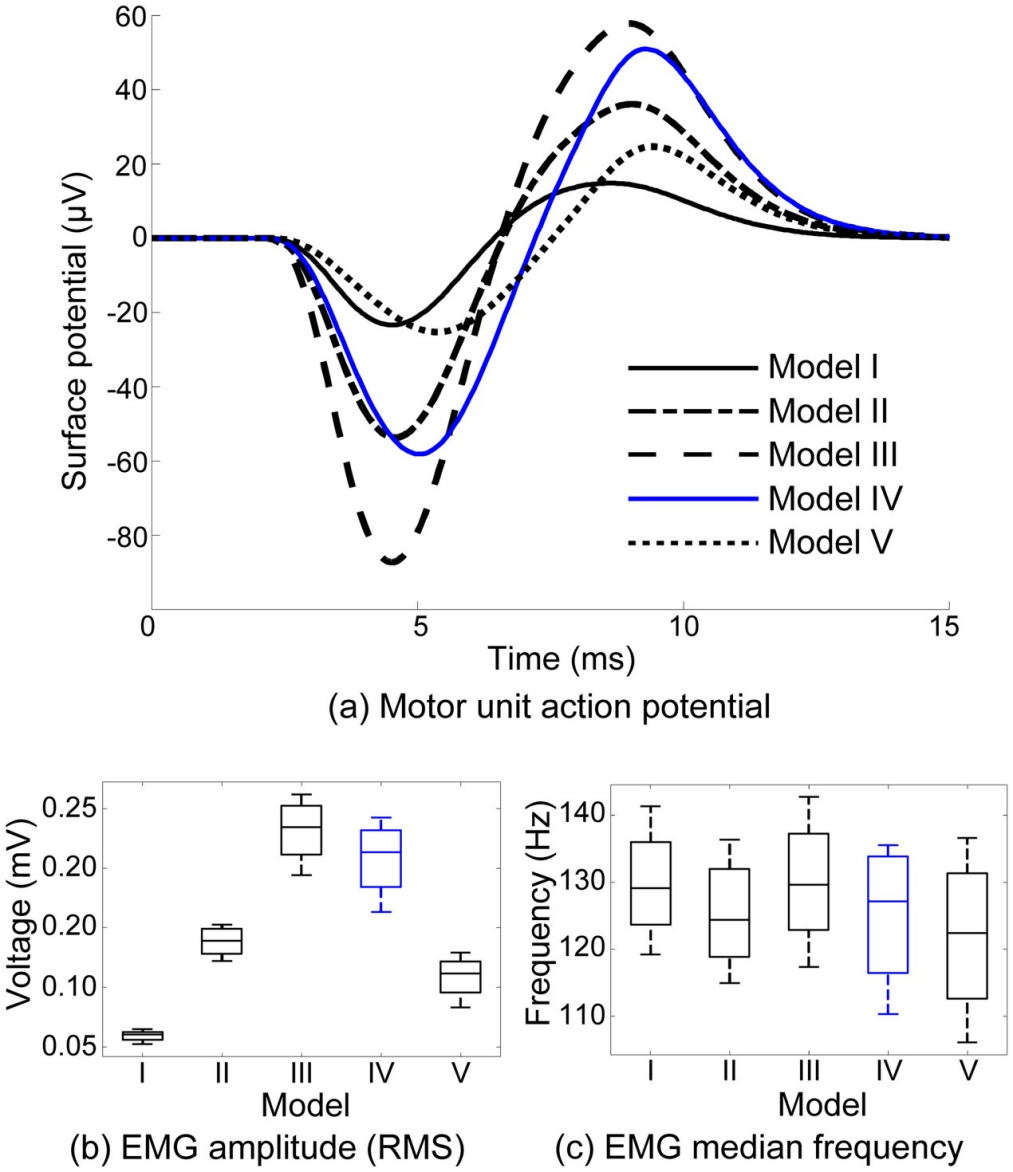
Simulated signals obtained from five distinct EMG models of varying geometrical and electrical characteristics. (a) Electric potential produced by the activation of a representative motor unit (MU 52) at a single monopolar electrode. Features of the electromyograms (monopolar channels) generated using the five models: RMS voltage (b) and median frequency (c). Model I—Analytical infinite volume conductor model (isotropic); Model II—Homogeneous FE model; Model III—Inhomogeneous FE model; Model IV—Inhomogeneous FE model with anisotropic FDI (anisotropy ratio 4.4); Model V—Inhomogeneous FE model with high FDI anisotropy (anisotropy ratio 10).

Models of increasing complexity were first compared to examine the influence of tissue geometrical and electrical properties on the surface detected MUAPs and the compound surface EMG signal. In moving from an infinite analytical EMG model (model I) to a homogenous FE model (model II), an increase in the simulated MUAP amplitude is observed due the inclusion of domain boundaries that constrain the diffusion of electric currents, producing higher currents and, consequently, higher voltages at the EMG electrode, Fig 3a.

An additional increase is observed when heterogeneity is considered (model III). This effect is similarly related to constraining of the current but is due here to the inclusion of tissues with relatively high resistivity: bone, fat, and skin. Since current is mostly confined to the muscle volume and the voltage drop across fat and skin is relatively low, the potential recorded by the electrode increases. In contrast, the inclusion of muscle anisotropy (model IV) leads to a reduction in signal amplitude. This effect becomes more evident as anisotropy ratio is increased (model V). Anisotropic models yielded smoother MUAPs, with lower frequency content, consistent with what has been previously observed in idealized models [56,14]. To assess these effects across the motor unit population, surface electromyograms were simulated using models I to V using independent sets of motoneuron firing times and four monopolar channels. The variations in RMS amplitude and EMG median frequency at the population level across the models corroborate the effects described for individual motor units, Fig 3b and 3c.

### FDI muscle fiber orientation differs during flexion and abduction and influences MUAP features

**Fig 4:**
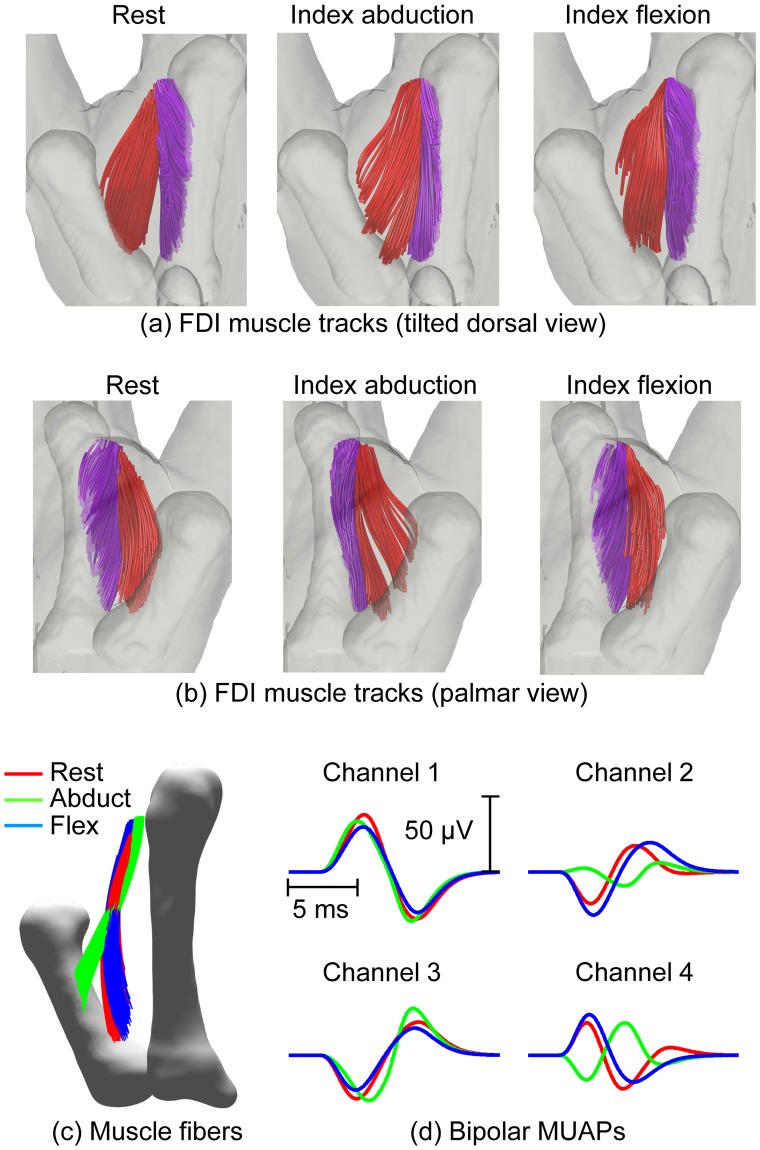
FDI muscle fiber tracks derived from the DWI data in three states: At rest, index finger abduction and index flexion and influence on simulated signals. DTI derived fiber tracks in the superficial head of the FDI are shown in red and in the deep head in pink: (a) palmar view (3D model tilted 35 degrees right) and (b) dorsal view. (c) Dorsal view (3D model tilted 10° left) of the FDI region displaying virtual muscle fibers corresponding to a representative motor unit (MU 52) under three conditions: rest (red), index finger abduction (green) and index flexion (blue). (d) Corresponding simulated motor unit action potentials (bipolar channels).

Clear differences in muscle fiber tracks curvature and direction were observed across the FDI muscle states, with fibers in the superficial head becoming “fanned” during abduction and fibers in both superficial and deep heads becoming more aligned with each other and with the direction of the 2^nd^ metacarpal during flexion, Fig 4a and 4b. The tracks shown in these figures represent the result of the DTI fiber tracking, rather than the virtual muscle fibers used in the simulations and shown in detail in Fig 2. The number of tracks identified, as well as the regions in which the tracks were estimated, varies depending on the specifics of the acquired diffusion data for each case, whereas virtual muscle fibers are generated in such a way that their number and distribution throughout the FDI cross-section remains constant across states.

The changes in fiber orientation and curvature influenced the corresponding simulated action potentials. While a relatively modest difference was observed between simulated MUAPs at rest and during flexion, more substantial changes were observed during abduction due to the more pronounced alterations in muscle architecture, Fig 4c and 4d. An inversion in polarity can also be observed at one electrode due to a shift in fiber orientation within the motor unit modifying the relative distances between fibers and electrode.

### Changes in fiber orientation and recruited motor units alter EMG amplitude and frequency content during flexion

**Fig 5:**
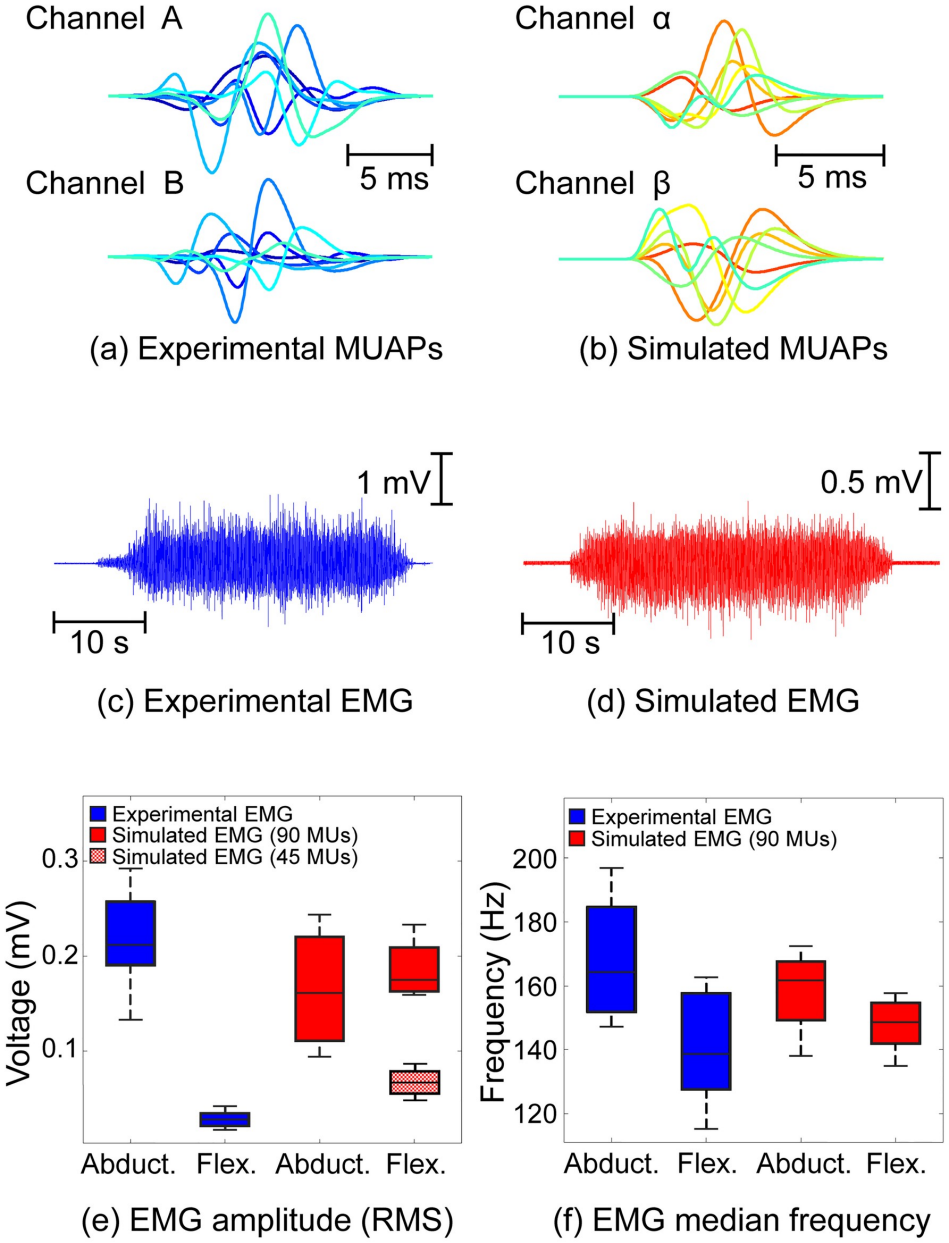
Experimentally recorded and simulated electrophysiological signals for the same subject. (a) Representative spike-triggered averaged motor unit action potentials for two bipolar channels decomposed by the EMG system. (b) Representative simulated motor unit action potentials from two bipolar channels. (c) Sample experimentally recorded electromyogram. (d) Sample simulated electromyogram. Experimental (blue) and simulated (red) EMG RMS voltages (e) and median frequencies (f). The dotted red box in (e) corresponds to the amplitudes of the simulated EMG signals when the population of recruited motor units was reduced to 50% (*i*.*e*., 45 motor units) of that considered in the other simulations (solid red boxes– 90 motor units).

Variability in the shapes of the simulated and experimentally recorded MUAPs compared well, Fig 5a and 5b. A good agreement was also observed between temporal and spectral features of the experimental and the synthetic EMG signals. The RMS amplitudes of the presented experimental and simulated EMG signals (Fig 5c and 5d) were 0.21 mV and 0.10 mV, respectively, with a skewness and kurtosis of 0.49 and 5.44, respectively, in the experimental, and 0.21 and 5.41 in the simulated signals.

There was a close agreement in the range of amplitudes of the simulated and experimental signals during abduction, with the median of the experimental RMS amplitudes lying within the inter-quartile range of the simulated signals (Fig 5e). Amplitudes of experimentally recorded EMG signals were lower during flexion (Fig 5e). A similar trend has been recently been reported [57] and attributed in part to a relatively smaller contribution of the FDI to force generation during index finger flexion, where extrinsic flexor muscles also contribute to force generation, than when acting as a primary abductor during abduction. While EMG activity confirmed activation of the FDI during flexion, the relative contribution of co-contracting extrinsic flexors here is not known. Ketchum *et al*. [58] estimated the relative contribution of intrinsic muscles to be 44% during a task which involved flexion of the metacarpophalangeal joint and simultaneous extension of the proximal and distal interphalangeal joints, similar to the protocol adopted in the present study. The population of active motor units during flexion was, therefore, reduced to 50% of the number active during abduction force of the same magnitude to simulate recruitment of a smaller population of FDI MUs during flexion, Fig 5e. EMG signals simulated with a reduced number of recruited MUs during flexion confirmed the experimentally observed trend of reduced EMG amplitudes with a narrower range, Fig 5e (dotted box). The reduction in the range of amplitudes, in both simulated and experimental data, is linked to the changes in muscle architecture, reflecting a closer alignment of fibers that arises when the muscle shifts from abduction to flexion (Fig 4a and 4b).

The EMG median frequency was also lower during flexion in both experimental and simulated EMG data (Fig 5f). In the experimental data, if fewer high threshold motor units are activated during flexion this effect may be partially explained by differences in the population of motor units recruited. However, the same trend was present in the frequency characteristics of the simulated signals, albeit to a slightly lesser extent, even when the population of contributing motor units remained constant, indicating that changes in muscle fiber direction and curvature in the different muscle states also contribute to this frequency shift.

### Directional anisotropy due to fiber orientation with motor unit size and location influences MUAP size and rate of decay

**Fig 6:**
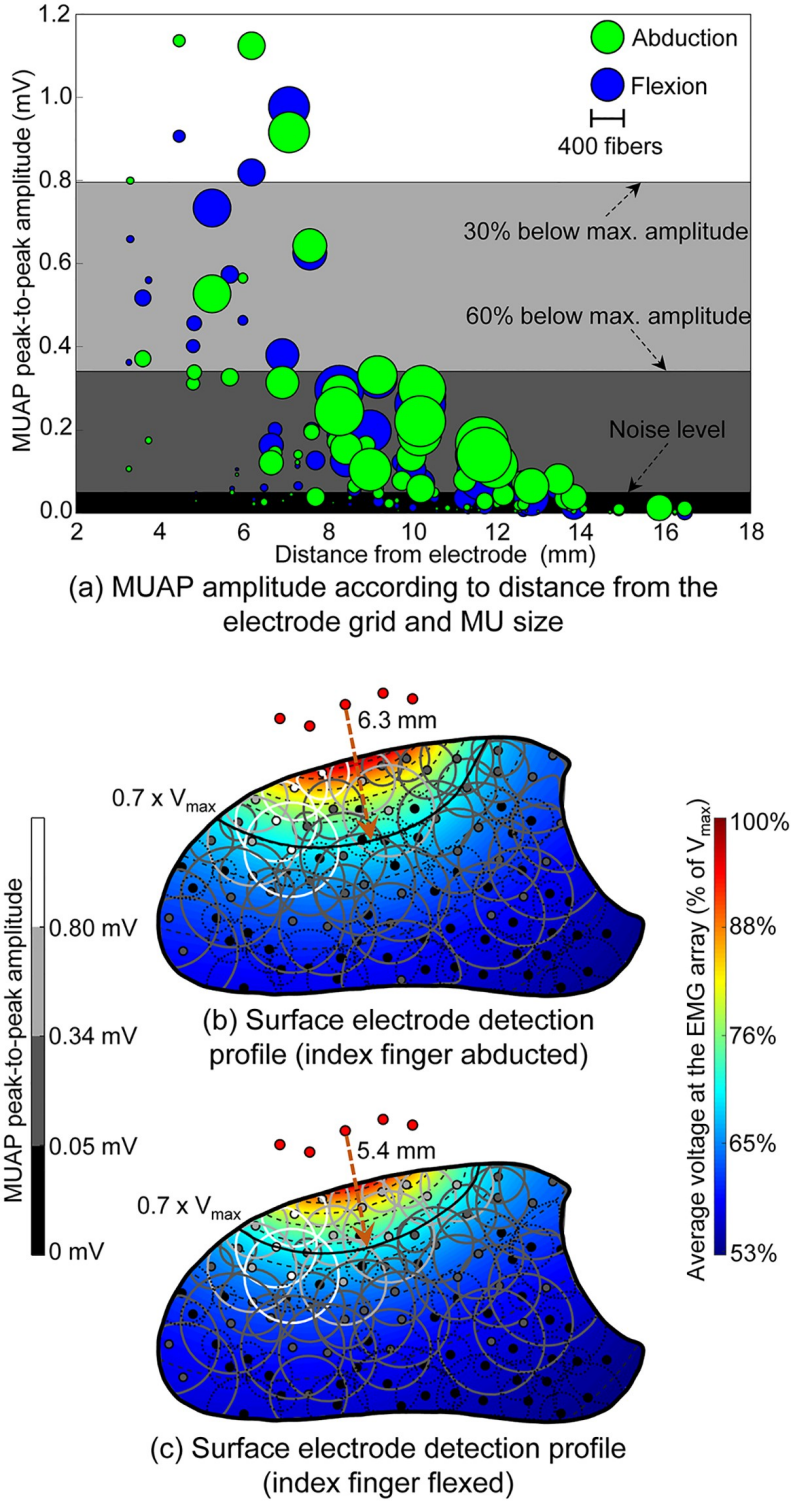
Influence of physiological and anatomical factors on the EMG amplitude. (a) MUAP peak-to-peak amplitude (maximum across four bipolar channels) as a function of distance from the electrode. The number of fibers in each motor unit is indicated by the diameter of the discs and the muscle state is indicated by their color: green for index finger abduction and blue for flexion. The electrical profile of the muscle over the cross-section directly under the electrode array is shown in (b) and (c) for abduction and flexion, respectively. This profile illustrates the normalized electric potential detected at the EMG array (averaged over the five electrodes) by virtual point current sources distributed over the muscle cross-section. The reference voltage V_max_ corresponds to the maximum voltage produced by these point sources across the two muscle states. Dashed lines mark levels of 5% decay in the voltage produced at the array. Center points and territories of simulated motor units are also shown. MUs for which the amplitude of their surface action potentials lay below the noise level are indicated by dotted black lines. Amplitudes of the surface action potentials for the remaining motor units are indicated by the color of the center point and the territory line, according to the color scale on the left side of the figure—same scale used in (a).

Finally, the model was used to examine the combined influence of motor unit size and location on the amplitude of the MUAPs detected at the skin surface during flexion and abduction, Fig 6. The largest amplitude action potentials were generated by larger MUs lying closest to the electrode, though relatively small superficial motor units lying very close also yielded similar amplitude MUAPs (Fig 6a). The amplitudes of a number of large motor units lying further from the electrode remains small. Of the 90 active MUs, the amplitude of 43 MUs (47.8%) lay above the noise level of 50 μV pk-pk and 4 MUs (4.4%) within 30% of the maximum MUAP amplitude observed, during abduction. During flexion, 45 MUs (50%) lay above the noise level while 3 MUs (3.3%) lay within 30% of the maximum MUAP amplitude. The differences in MUAP amplitude across muscle states results from a combination of changes in fiber arrangement and the consequent changes in muscle tissue electrical anisotropy. By comparing Fig 6b and 6c, one can observe the motor unit territories, their distances from the electrodes, and how the resulting MUAP amplitudes change, depending on the muscle state.

**Fig 7:**
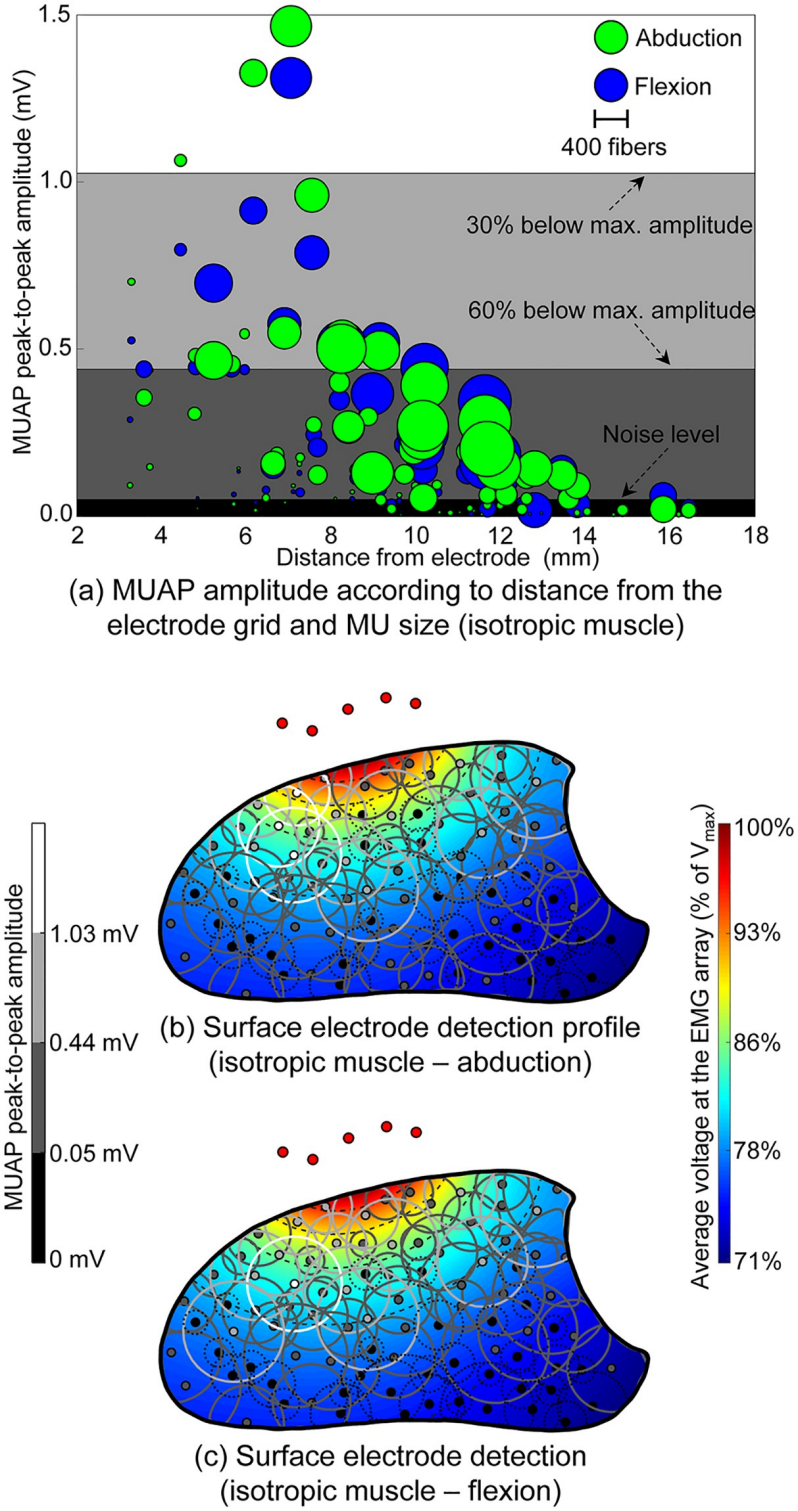
Influence of physiological and anatomical factors on EMG amplitude when muscle anisotropy is neglected. Data are as presented in Fig 6. (a) MUAP peak-to-peak amplitude as a function of distance from the electrode. The number of fibers in each motor unit is indicated by the diameter of the discs and the muscle state is indicated by color. The electrical profile of the muscle over the cross-section directly under the electrode array is shown in (b) and (c) for abduction and flexion, respectively. The reference voltage V_max_ corresponds to the maximum voltage produced by these point sources across the two muscle states. Dashed lines mark levels of 5% decay in the voltage produced at the array. MUs for which the amplitude of their surface action potentials lay below the noise level have their territories indicated by dotted black lines. The remaining MUs have their surface AP levels indicated by the color of the center point and the territory line, according to the color scale given at the left side of the figure.

The electrical profile of the muscle is also presented alongside the MUAP amplitude in Fig 6b and 6c, for abduction and flexion, respectively. This describes the relative electric potential produced at the electrodes by current sources located throughout the muscle cross-section, illustrating the impact of changes in geometrical and electrical properties of the tissue without the confounding effects of motor unit size, fiber type and action potential trajectories. A compression in the pick-up area of the electrode when switching from abduction to flexion is observed, with the radius of the region where the potential decays to 30% of the maximum value across states decreasing from 6.3 mm to 5.4 mm. This effect was found to be due to changes in the direction of the anisotropy of the muscle tissue: during abduction, fibers tended to project from the dorsum to the palm of the hand at the cross-section region close to the electrode array, whereas fiber alignment tended to remain more parallel to the skin surface during flexion (Fig 2c and 2d). From an electrical point of view, the fiber arrangement during flexion yields a decrease in the apparent tissue conductivity in the direction into the muscle, leading to a lower electric potential with a sharper decay rate in the vicinity of the EMG array, when compared to the abduction state. Differences between electrical profiles across muscle states cannot be observed when muscle anisotropy is neglected (Fig 7).

## Discussion

The emergence of imaging techniques that enable anatomical structures to be visualized *in vivo* provides new opportunities for the refinement of physiological models. Advances in EMG acquisition and processing are simultaneously moving towards increasingly accurate identification of motor unit activity under challenging conditions such as dynamic contractions, which require ever more sophisticated understanding of the biophysics of EMG generation. It is widely accepted that the electrical and geometrical properties of the surrounding tissues substantially influence the MUAP waveform properties. However, few models have attempted to capture these in a realistic manner. In the models that have constructed the surrounding volume conductor from MRI data, the computational complexity of solving for the electrical field over time, as action potentials propagate along individual muscle fibers, has limited the application of such models to simulation of a small number of fibers [17,18]. Furthermore, incorporation of realistic muscle fibers trajectories and tissue anisotropy, and the manner in which they change during muscle contraction, has not yet been addressed. To capture these effects, we have combined diffusion tensor imaging methods with electromagnetic modelling of the extracellular potential and a model of the motoneuron pool. This allows us to simulate subject-specific single muscle fiber action potentials, motor unit action potentials and the interference EMG signal from the FDI muscle during flexion and abduction.

To overcome the challenge of the computational complexity in the electromagnetic modelling, while retaining the physiological and anatomical detail, we make use of the principle of reciprocity. Used here for the first time in simulation of EMG, this principle requires a reduced number of finite element model solutions for an arbitrary number of muscle fibers. In our approach, the distribution of the electric potential is evaluated once for each electrode rather than for each muscle fiber at every time point during action potential propagation. As a result, there is a substantial gain in computational efficiency when compared to classical approaches, enabling large numbers of muscle fibers to be simulated with very low computational cost. Examples are presented here for bipolar signals obtained using a five-sensor array, but the approach can be extended to any configuration including high density arrays comprised of large numbers of electrodes.

The application of DTI to the intrinsic muscles of the hand enables us to visualize for the first time the complex structure of the FDI muscle and the changes in fiber arrangement that occur *in vivo*. DTI fiber tracking revealed the distinct superficial and deep heads of the muscle, which can be visually identified in cadaveric specimens [59]. A close agreement in terms of muscle fiber architecture was observed between the obtained tracks and the cadaveric reference (Fig 1b and 1c), with the fiber orientation and curvature in the superficial and deep heads of the FDI similar in both. The location of the muscle aponeurosis in the model, as estimated from the region of convergence of the fiber tracks from the superficial and deep heads, was also found to agree with the cadaveric specimen.

DTI fiber tracking further revealed changes in the muscle fiber orientation and curvature during index finger abduction and flexion which have not been previously reported (Fig 4a and 4b). These changes may result directly from activation of the muscle fibers, indirectly as a result of a change in joint orientation, or from a combination of both. In the present study, it is not possible to separate the relative contribution of both factors. The MRI-compatible rig was designed to limit the displacement of the index finger around the MP joint to a 10 degrees angle. In addition, activation of the FDI muscle was confirmed by EMG activity recorded during both abduction and flexion. It is thus unlikely that the displacement of the index finger alone is responsible for the observed changes in muscle fiber arrangement but rather these are a consequence of both muscle activation and any concurrent changes in joint orientation.

While DTI provides detailed information on the orientation of fibers within the muscle, little information is available on the shape of the motor unit territories across the different states. In the absence of precise information on the shape, distribution and overlap of individual motor unit territories, the assumption of uniform fiber distribution with circular motor unit territory represents an approximation to the physiological distribution. Differences in the shape and distribution of the shape motor unit territories may further influence the morphology of the corresponding motor unit action potentials and can be readily incorporated into the model as this information becomes available. Similarly, in the absence of information on how motor unities cross-sectional territories change from rest to abduction and flexion, their locations and boundaries in the reference plane were assumed to remain fixed across states. As a consequence, in some cases the simulated fibers origins and insertions of a given motor unit may change slightly according to the change in fiber trajectories. Nevertheless, the changes in fiber direction and curvature arising from index abduction and flexion as identified by the DTI fiber tracking are captured by the model and are the primary cause of the observed differences in EMG features across states.

Analyses of EMG features under simulated abduction and flexion conditions further revealed differences in amplitude and frequency content consistent with those observed experimentally (Fig 5f). The simulations revealed that differences in the FDI muscle fiber architecture between abduction and flexion result not only in a change in the amplitude of the detected motor unit action potentials, but in the effective pick-up volume of the electrode and in the population of detectable motor units (Fig 6b and 6c).

Estimating the pick-up or detection volume of the surface EMG electrode is a fundamental question in interpreting features of signals recorded at the skin surface. It is well-established that detection volume is influenced by factors including inter-electrode distance, subcutaneous fat thickness, tissue anisotropy and proximity to tissues with conductivities higher or lower than that of muscle, such as bone or blood vessels [5,7,14,60]. Detection volume increases with increasing inter-electrode distance and subcutaneous fat thickness, as deeper motor units contribute relatively more to the surface EMG signal [7,60]. The region from which 95% of the EMG RMS amplitude originates has been estimated to lie within 7–8 mm of the electrode in an idealized model of the arm for a similar inter-electrode distance and subcutaneous fat thickness to the present model [60]. Earlier analytical models have estimated the surface electrode detection depth to be within a similar range, approximately equal to the inter-electrode distance [61] or 8 mm for a 11 mm interelectrode distance [7], in a uniform population of muscle fibers or motor units, respectively. In the present study, motor units generating MUAPs with amplitudes greater than 60% of the maximum amplitude of the largest MUAP were concentrated within a region approximately 7 mm from the electrode, extending a distance of 4–5 mm into the muscle, Fig 6. The differences between the estimated decay rate in the present study can be accounted for by the precise electrical and geometrical properties of the surrounding tissues in the inhomogeneous model. A faster rate of decay of MUAP amplitude with increasing distance from the electrode was also observed during flexion than during abduction, due to the change in tissue anisotropy with fiber orientation across states (Fig 6a, 6b and 6c). Furthermore, by incorporating a distribution of motor unit sizes and types, the contribution of both size and location to the surface-detected MUAP can be simultaneously examined.

Understanding the contribution of constitutive, anatomical and physiological properties of muscle to the generation of the electromyogram is essential in enabling accurate, physiologically-relevant information to be extracted from the signal. This is particularly true under dynamic conditions, where muscle fiber arrangement and surrounding tissue morphologies change during muscle contraction. As EMG acquisition and analysis techniques move towards the identification of individual motor unit activity during dynamic contractions, advanced electrophysiological models that incorporate emerging data on muscle fiber architecture during activation, such as that introduced here, are required. These models also constitute a fundamental basis for the development of new techniques that will use electrophysiological data to infer anatomical information about the organization of motor units within the muscle.

## Supporting information

S1 Fig Experimental conditions under which EMG signals and MRI data were recorded. (a) MRI-compatible rig. (b) Index finger abduction. (c) Index finger flexion. The angular displacement of the index finger was 10° and the applied force was 6.5 N for both abduction and flexion contractions. The red circles indicate the position of the electrode array utilized to record the subject’s electromyogram.
(TIF)
