# Supplementary figures and images for: Anatomically accurate model of EMG during index finger flexion and abduction derived from diffusion tensor imaging
Source: PLoS Comput Biol. 2019 Aug 29;15(8):e1007267. doi: 10.1371/journal.pcbi.1007267 (PMC6738720; doi:10.1371/journal.pcbi.1007267)

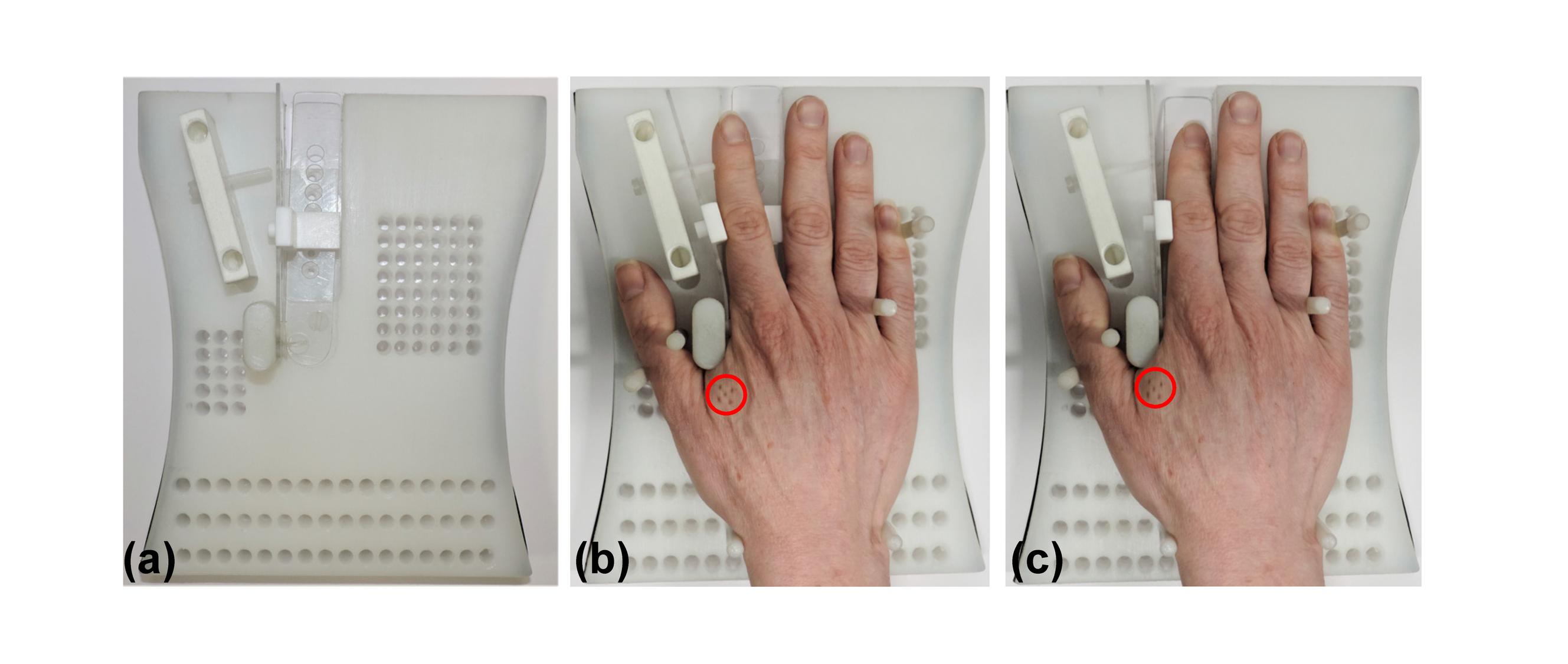

Supplement: S1 Fig — (a) MRI-compatible rig. (b) Index finger abduction. (c) Index finger flexion. The angular displacement of the index finger was 10° and the applied force was 6.5 N for both abduction and flexion contractions. The red circles indicate the position of the electrode array utilized to record the subject’s electromyogram. (TIF) [file pcbi.1007267.s001.tif]
